# Leadless Pacemaker Implantation, Focusing on Patients With Conduction System Disorders Post–Transcatheter Aortic Valve Replacement: A Retrospective Analysis

**DOI:** 10.1016/j.cjco.2023.10.009

**Published:** 2023-10-14

**Authors:** Julius Jelisejevas, François Regoli, Daniel Hofer, Giulio Conte, Tardu Oezkartal, Ardan M. Saguner, Maria Luce Caputo, Lorenzo Grazioli, Jan Steffel, Angelo Auricchio, Alexander Breitenstein

**Affiliations:** a Department of Cardiology, University Hospital Zurich, Zurich, Switzerland; b Fondazione Cardiocentro Ticino, Lugano, Switzerland; c Ospedale Regionale di Bellinzona e Valli, Bellinzona, Switzerland

## Abstract

**Background:**

Impairment of the conduction system is a common complication of transcatheter aortic valve replacement (TAVR), which is typically performed in elderly patients. A leadless pacemaker (LP) may be a suitable option in this frail population, but the available scientific data concerning the efficacy and safety of leadless pacing after TAVR are sparse. The purpose of this analysis was to evaluate the efficacy and safety of LP implantation in patients with relevant bradycardias after TAVR, compared to other indications.

**Methods:**

Consecutive patients were retrospectively enrolled. Demographics, background heart diseases, interventional parameters, and follow-up data were collected.

**Results:**

A total of 257 consecutive patients who underwent LP implantation were included. In 26 patients, the device was implanted due to bradycardias after TAVR (TAVR group), whereas the remaining 231 patients were in the population without previous TAVR (non-TAVR group). The mean implantation duration (56 ± 22 minutes in the TAVR group vs 48 ± 20 minutes in the non-TAVR group; *P* = not significant [NS]) and the implantation success rate (100% in the TAVR group vs 98.7% in the non-TAVR group; *P* = NS) were similar in the 2 cohorts. No significant differences occurred in pacing parameters (sensing, impedance, and threshold, respectively) between the 2 groups, either at implantation or during follow-up. A total of 8 major periprocedural complications (3.1% of patients in total; 3.8% in the TAVR group vs 3.0% in the non-TAVR group; *P* = NS) occurred within 30 days, without significant difference between the 2 groups.

**Conclusions:**

LP implantation appears to be safe and effective in patients after TAVR, and therefore, this procedure is a suitable option for this often old and frail population.

## Résumé

**Contexte:**

L’atteinte du système de conduction cardiaque est une complication courante du remplacement valvulaire aortique par cathéter (RVAC), une intervention habituellement pratiquée chez les patients âgés. Un stimulateur cardiaque sans sonde peut être une option convenable pour cette population fragile, mais les données scientifiques actuelles concernant l’efficacité et l’innocuité de la stimulation sans sonde après un RVAC sont fragmentaires. Cette analyse visait à évaluer l’efficacité et l’innocuité de l’implantation d’un stimulateur cardiaque sans sonde chez des patients atteints de bradycardies pertinentes après un RVAC, comparativement à d’autres indications.

**Méthodologie:**

Des patients consécutifs ont été recrutés de manière rétrospective. Les données démographiques, les maladies cardiaques sous-jacentes, les paramètres interventionnels et les données de suivi ont été colligés.

**Résultats:**

Un total de 257 patients consécutifs qui se sont fait implanter un stimulateur cardiaque sans sonde ont été inclus. Chez 26 patients, le dispositif a été implanté en raison d’une bradycardie après un RVAC (groupe RVAC), alors que les 231 autres patients formaient la population sans RVAC antérieur (groupe sans RVAC). La durée moyenne de l’intervention d’implantation (56 ± 22 minutes dans le groupe RVAC *vs* 48 ± 20 minutes dans le groupe sans RVAC; *p* = non significatif [NS]) et le taux de réussite de l’implantation (100 % dans le groupe RVAC *vs* 98,7 % dans le groupe sans RVAC; *p* = NS) étaient similaires dans les deux cohortes. Aucune différence significative n’a été observée dans les paramètres de stimulation (sensibilité, impédance et seuil, respectivement) entre les 2 groupes, que ce soit au moment de l’implantation ou pendant le suivi. Un total de 8 complications périopératoires majeures (3,1 % de l’ensemble des patients; 3,8 % dans le groupe RVAC *vs* 3,0 % dans le groupe sans RVAC; *p* = NS) sont survenues dans les 30 jours, sans différence notable entre les 2 groupes.

**Conclusions:**

L’implantation d’un stimulateur cardiaque sans sonde semble sûre et efficace après un RVAC; par conséquent, cette intervention représente une option convenable pour cette population souvent âgée et fragile.

Transcatheter aortic valve replacement (TAVR) has become a standard in treating aortic stenosis in patients at intermediate to high surgical risk, a subset that often includes elderly and frail patients.^1^^,^^2^ TAVR-related conduction disturbances requiring permanent pacemaker implantation remain the most common complication, with a risk level of 9%-30%, depending on the implantation technique and type of device.^3^^,^^4^ Conventionally, lead-driven transvenous pacing systems have been the preferred approach when permanent pacing was required. However, conventional pacemakers carry relatively high short- and long-term complication rates with an equally high incidence of morbidity and mortality, including lead malfunction and infection.^5^ Leadless pacemakers (LPs) were developed to overcome potential complications of conventional lead-driven transvenous devices, especially in elderly and frail patients. The safety and efficacy of the currently approved LP (Micra Transcatheter Pacing System, Micra TPS, Medtronic, Minneapolis, MN)^6^ have been demonstrated in a nonrandomized, prospective study and were confirmed in a large retrospective registry.^7^^,^^8^ However, the available data concerning the efficacy and safety of LPs after TAVR are limited, and therefore, obtaining more information on the efficacy and safety of leadless pacing in this patient population is of high importance.

## Material and Methods

### Study population and data collection

A total of 257 consecutive patients who underwent Micra TPS implantation at 2 high-volume centres in Switzerland (University Hospital Zurich, Zurich and Cardiocentro Ticino Institute, Lugano) between June 2015 and March 2021 were included in this retrospective 2-centre cohort study. The vast majority of the patients received a Micra TPS without an atrioventricular (AV) synchrony function (Micra AV), owing to the limited availability during the study period. The study was approved by the local ethics committee (KEK-ZH-Nr: 2020-00811). All enrolled patients had a clinical indication for a single-chamber pacemaker and provided written informed consent to undergo the procedure and participate in the study.

### LP implantation

LPs (Micra TPS) were implanted according to the manufacturer’s recommendation and as previously described.^9^ All implantations were performed in the catheterization laboratory under conditions of local anesthesia and conscious sedation if this was deemed necessary. Briefly, after gaining femoral venous access, a super-stiff wire is advanced into the superior vena cava. After predilatation of the access site, the 27-Fr Micra TPS introducer sheath is advanced into the right atrium. Using this access, the Micra TPS delivery tool, together with the device, is advanced into and positioned in the right atrium. After crossing the tricuspid valve, the device is placed into the septal wall of the right ventricle. When adequate fixation of the device tines by the pull-and-hold test is confirmed, the electrical parameters are tested. If these are within the acceptable range, the tether is cut and pulled out. Hemostasis is assured via a modified figure-of-8 stitch or a Perclose ProGlide device (Abbott Medical, USA) in all cases.

### Definition of major periprocedural complications

Major procedure-related complications were defined according to previous publications, and these included complications prolonging hospital stay, requiring readmission, or resulting in significant disability or death within 30 days as a result of device implantation.^10^^,^^11^ Bleeding was defined as a major complication if it required treatment with transfusion or resulted in a 20% or greater fall in hemoglobin level. A major vascular complication was defined as one that required intervention, including surgical repair or transfusion, prolonged the hospital stay, or required hospital admission. Other major complications included pericardial effusion in need of pericardiocentesis or surgical treatment, permanent loss of device function (capture thresholds, pacing impedance, sensing), device-revision within 30 days, infection, device dislodgement, severe damage to the tricuspid valve, and relevant femoral hematoma needing intervention.

### Statistical analysis

Procedural characteristics and outcomes data were reviewed and collected from electronic medical records. Statistical analysis was performed using the Jamovi Project (2020; Jamovi Version 1.2), retrieved from https://www.jamovi.org. Normality of distribution was assessed for all variables using a Shapiro-Wilk test. Continuous variables were presented as mean ± standard deviation (SD), for normally distributed values, or as median (interquartile range [IQR]) for non-normally distributed values. Categorical variables were presented as counts (%). Comparisons between variables were performed using a Student *t* test, a Mann-Whitney *U* test, a χ^2^ test, or a Fisher exact test, as appropriate. A 2-tailed *P* < 0.05 was considered statistically significant.

## Results

### Study population and baseline characteristics

Of 257 consecutive patients who underwent LP implantation, 26 patients were implanted after undergoing TAVR (TAVR group), and 231 were implanted for other underlying diseases (non-TAVR group; Table 1). In the TAVR group, 4 patients were identified as having right bundle branch block prior to TAVR and as being patients at high risk for complete AV block. Mean age at implantation (82.0 ± 5.6 years in the TAVR group vs 81.1 ± 9.0 years in the non-TAVR group; *P* = not significant [NS]) and baseline characteristics were equally distributed, except for chronic kidney disease being more common in the TAVR group (77% in the TAVR group vs 52% in the non-TAVR group; *P* = 0.017). Atrial fibrillation with complete AV block was more often the indication for LP implantation in the TAVR group (46% in the TAVR group vs 9% in the non-TAVR group; *P* < 0.001; Table 2), whereas atrial fibrillation with slow conduction was the predominant reason for LP implantation in the non-TAVR group (8% in the TAVR group vs 39% in the non-TAVR group; *P* = 0.002). In the TAVR group, the median duration between valve intervention and LP implantation was 8 days (range: 0-368 days). In most of the cases (88.5%), an LP was implanted very early (at < 7 days) or early (at 7-30 days) after the TAVR procedure (Table 3).

**Table 1:** Baseline characteristics of the patient population

Characteristic	All patients (n = 257)	TAVR group (n = 26)	Non-TAVR group (n = 231)	*P*
Age, y	81.1 ± 8.7	82.0 ± 5.6	81.1 ± 9.0	0.498
Male	167 (65)	16 (62)	151 (65)	0.471
LVEF, %	55.5 ± 10.0	52.3 ± 13.5	55.9 ± 9.5	0.193
Underlying heart disease				
Coronary artery disease	94 (37)	17 (65)	77 (33)	0.517
Valvular disease	72 (28)	26 (100)	48 (21)	0.001
Comorbidities				
Chronic renal failure	141 (55)	20 (77)	121 (52)	0.017
Hemodialysis	7 (2.7)	0 (0)	7 (3)	0.359
Peripheral artery disease	24 (17)	6 (23)	30 (13)	0.160
COPD	35 (14)	2 (8)	33 (14)	0.353
Diabetes mellitus	50 (19)	8 (31)	42 (18)	0.124
Prior stroke	32 (12)	4 (15)	28 (12)	0.633
Prior pulmonary embolism	7 (3)	1 (4)	6 (3)	0.711
Cancer	40 (16)	5 (19)	35 (15)	0.586
Prior pacemaker	24 (9)	0 (0)	24 (9)	0.084
Cardiogenic shock	10 (4)	1 (4)	9 (4)	0.990

Values are mean ± standard deviation, or n (%), unless otherwise indicated.

COPD, chronic obstructive pulmonary disease; LVEF, left ventricular ejection fraction; TAVR, transcatheter aortic valve replacement.

**Table 2:** Pacing indication of the patient population

Pacing indication	All patients (n = 257)	TAVR group (n = 26)	Non-TAVR group (n = 231)	*P*
AA				
Slow AA	92 (36)	2 (8)	90 (39)	0.002
AA and complete AV block	32 (12)	12 (46)	20 (9)	< 0.001
Tachy-brady syndrome	42 (16)	1 (4)	41 (18)	0.069
SR				
Postconversion pause	11 (4)	1 (4)	10 (4)	0.908
Sinus node dysfunction	18 (7)	1 (4)	17 (7)	0.506
SR with complete AV block	42 (16)	4 (15)	38 (16)	0.889
Other causes∗	20 (8)	5 (19)	15 (6)	0.022

Values are n (%), unless otherwise indicated.

AA, atrial arrhythmia; AV, atrioventricular; SR, sinus rhythm; TAVR, transcatheter aortic valve replacement.

∗ Other causes are left bundle branch block post-TAVR; cardioinhibitory response; syncope prevention, right bundle branch block and left anterior hemiblock.

**Table 3:** Days between transcatheter aortic valve replacement (TAVR) and leadless pacemaker (LP) implantation

TAVR to LP implantation (n = 26), d	Value, n (%)
Very early (≤ 7)	13 (50)
Early (> 7, ≤ 30)	10 (38.5)
Late (> 30, ≤ 90)	1 (3.8)
Very late (> 90)	2 (7.7)

In the non-TAVR group, 24 patients had a prior implanted conventional (transvenous or epicardial) pacemaker system; 20 of these cases required complete extraction of the former system due to infection, malfunction, or lead perforation. The remaining 4 cases did not require extraction of the conventional system.

### Procedure characteristics

The mean implantation procedure duration (56.2 ± 22.3 minutes in the TAVR group vs 48.2 ± 20.3 minutes in the non-TAVR group; P = NS) and implantation success rate (100.0% in the TAVR group vs 98.7% in the non-TAVR group; P = NS) were similar between the 2 groups. In 2 patients, the LP implantation was unsuccessful due to tortuous venous anatomy, and in one patient, it was unsuccessful due to unacceptable device parameters during implantation. The final LP position was the mid-septal area of the right ventricle in the majority of patients in both cohorts, without a statistical difference (76.7% in the TAVR group vs 57.1% in the non-TAVR group; *P* = NS). Radiation exposure time was similar between the groups (7.6 ± 5.6 minutes in the TAVR group vs 7.8 ± 8.0 minutes in the non-TAVR group; P = NS; Table 4). Left femoral access for LP implantation was used more often in the TAVR group (30.8% in the TAVR group vs 5.2% in the non-TAVR group; *P* < 0.012), with an excellent implantation success rate and without statistically significant differences in procedure and fluoroscopy times (Table 5).

**Table 4:** Implantation characteristics

Characteristic	All patients (n = 257)	Post-TAVR (n = 26)	Non-TAVR (n = 231)	*P*
Capture threshold, V/0 24 ms	0.57 ± 0.32	0.49 ± 0.16	0.59 ± 0.34	0.019
Sensing, mV	10.7 ± 5.0	10.2 ± 4.5	10.7 ± 5.1	0.651
Impedance, O	731 ± 165	709 ± 126	733 ± 171	0.382
Procedure time, min	49.0 ± 20.6 (42)	56.2 ± 22.3 (50.5)	48.2 ± 20.3 (40)	0.093
Fluoroscopy time, min	7.7 ± 7.8	7.6 ± 5.6	7.8 ± 8.0	0.265

Data are presented as mean ± standard deviation (median).

Values of capture threshold, sensing, and impedance are at time of implantation.

TAVR, transcatheter aortic valve replacement.

**Table 5:** Procedure characteristics in patients with transfemoral transcatheter aortic valve replacement history

Characteristic	Left femoral access (n = 8)	Right femoral access (n = 18)	*P*
Procedure time, min	47.5 ± 15.1	61.6 ± 23.0	0.079
Fluoroscopy time, min	7.23 ± 4.9	6.8 ± 4.1	0.829

Data are presented as mean ± standard deviation.

### Device parameters at implantation

**Figure 1:**
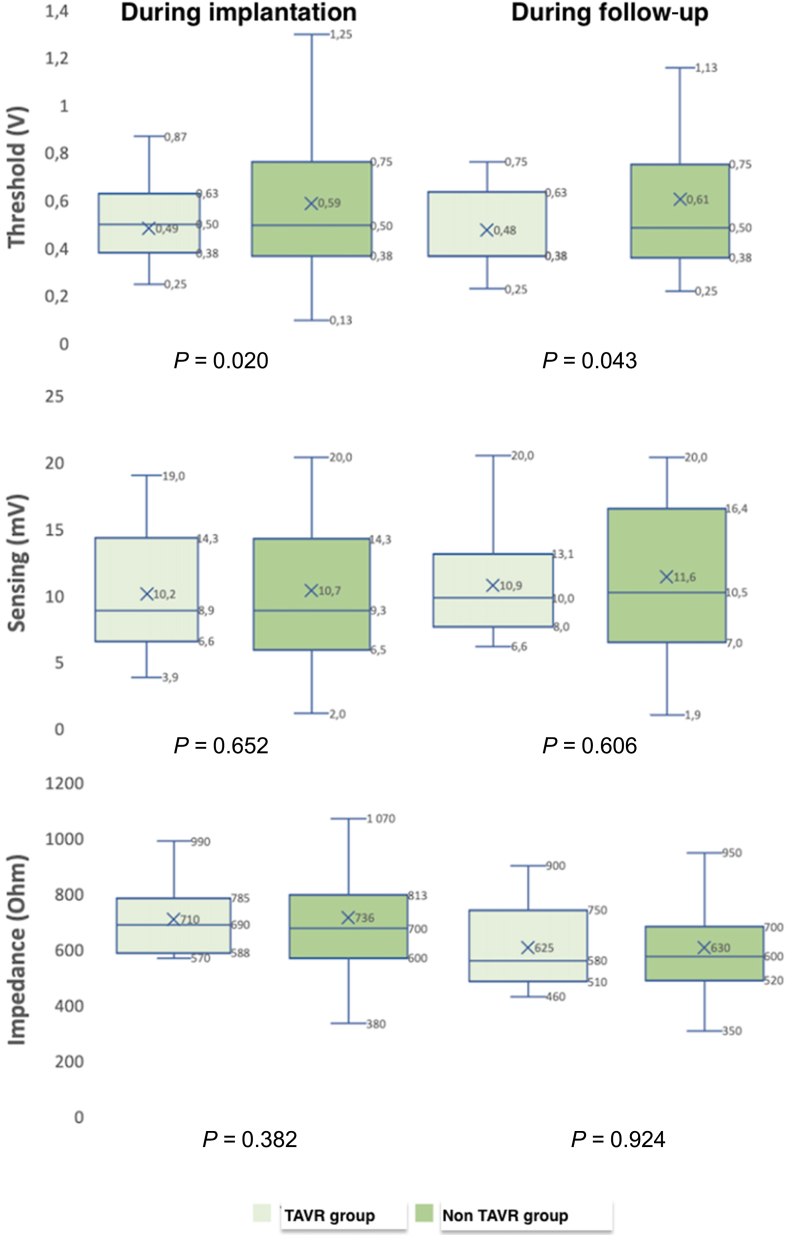
Device parameters for the patient population with previous transcatheter aortic valve replacement (TAVR; n = 26) group vs non-TAVR group at implantation and last follow-up.

The average pacing threshold at implantation (always at a pulse duration of 0.24 ms) for the entire population was 0.57 ± 0.32 V, with a right ventricular (RV) sensing of 10.7 ± 5.0 mV and an impedance of 731 ± 165 O (Table 4). Ventricular sensing and device impedance did not differ between the groups (Table 4; Fig. 1), whereas a statistically, but not clinically, significant difference occurred in the pacing threshold at implantation (0.49 ± 0.16 V in the TAVR group vs 0.59 ± 0.34 V in the non-TAVR group; *P* = 0.019; Table 4).

### Complications at implantation and during the 30-day follow-up

A total of 8 major complications (3.1%) occurred within 30 days after LP implantation (Table 6), without a statistical difference between the groups (3.8% in the TAVR group vs 3.0% in the non-TAVR group; P = NS). Four pericardial effusions occurred, of which one was managed conservatively, and 3 required a drainage, but none required cardiac surgery. One of these pericardial effusions occurred in the TAVR group and required pericardiocentesis, which was the only major complication in this group. Relevant femoral bleeding was encountered in 3 patients (1 had arterial bleeding with subsequent covered stenting of the femoral artery; 2 had venous bleedings that were managed successfully conservatively). One occurrence of intra-abdominal bleeding of unknown origin was noted in a patient with pericardial effusion, and both issues were managed conservatively. In the 30-day postinterventional period, 1 death, unrelated to LP implantation, occurred in the non-TAVR group.

**Table 6:** Major complications in 30-day postoperative period

Characteristic	All patients (n = 257)	Post-TAVR (n = 26)	Non-TAVR (n = 231)
Total major complications	8 (100)	1 (100)	7 (100)
Pericardial effusion∗	4 (50)	1 (100)	3 (43)
Femoral bleeding	3 (37.5)	0 (0)	3 (43)
Intra-abdominal bleeding†	1 (12.5)	0 (0)	1 (14)
Device dislodgement	0 (0)	0 (0)	0 (0)
Device infection	0 (0)	0 (0)	0 (0)

Values are n (%). Because of small numbers, *P* values could not be calculated reliably.

TAVR, transcatheter aortic valve replacement.

∗ Managed either conservatively or with pericardiocentesis.

† Treated conservatively.

### Midterm follow-up

During midterm follow up, the average device parameters (threshold, sensing, impedance) remained stable and were similar between the 2 groups (Fig. 1). However, 2 devices, both in the non-TAVR group, were successfully extracted percutaneously during follow-up (one extraction was due to elevated capture thresholds and a depleted battery after 23 months, with subsequent implantation of a conventional transvenous pacing system^12^; the other was due to loss of capture 4 months after device implantation). During a median follow-up period of 334 days (range: 10-1094 days), 22 deaths occurred (8.6% of all patients), 3 of them in the post-TAVR group; none of these deaths were related to the LP implantation procedure or the device itself. The average left ventricular ejection fraction (LVEF) at last follow-up was 54%, with no significant difference between the 2 groups.

## Discussion

LP implantation for bradyarrhythmias after TAVR demonstrated a high procedural success rate and a low number of complications in our retrospective study. An important finding is that, despite the fact that the TAVR population usually suffers from more comorbidities and hence represents a sicker patient group, we observed no significant difference regarding device efficacy, safety, or performance, compared to LP implantation without a previous TAVR.

### Efficacy and safety of leadless pacing after TAVR

We were able to demonstrate a very high success rate of LP implantation in the TAVR and non-TAVR groups, without a significant difference (100% vs 98.8%), which correlates with the data from the Micra TPS investigational device exemption (IDE) study [7] and the Post-Approval Registry [9]. Furthermore, other reports comparing LP vs transvenous pacemakers in patients after TAVR have shown comparable performance of both systems, suggesting that an LP might be a reasonable alternative in post-TAVR patients when single-chamber pacing is indicated.^13^ Even though a left-sided femoral access was chosen more often in our cohort in the early post-TAVR group, it did not affect the implantation success rate, a result that is also in line with a previous study.^14^ These data indicate that left-sided access is feasible, produces results comparable to those with a right-sided approach, and can be used as an alternative. More importantly, TAVR can result in a decline of right ventricular function, including worsening of tricuspid regurgitation,^15^ both of which can negatively impact the LP implantation success rate. Although RV function and tricuspid regurgitation were not assessed in the context of this analysis, the high implantation success rate in the TAVR population indicates that implanting an LP in patients suffering from impaired RV function, which would require such success, likely also will be possible.

The rate of major periprocedural complications within 30 days of LP implantation in the TAVR group was 3.8%, which seems to be slightly higher compared to results from the large IDE registry, in which a major complication rate of 4.0% was seen after 6-months of follow-up.^16^ However, no significant difference was present in the complication rate for the TAVR group compared to the non-TAVR group, and the difference in complication rate between this cohort and that in previous reports of a general LP population needs to be interpreted in the context of the usually frailer TAVR population. Indeed, the present TAVR cohort is older (82.0 ± 5.6 years) compared to the population in the IDE registry (75.9 ± 10.9 years), but it is in line with previous reports of TAVR patients who underwent LP implantation.^17^ The finding of comparable outcome results to those of these patients underscores the more fragile nature of this population. More specifically, the single major complication observed in this TAVR group, which was a pericardial effusion necessitating drainage, resulted from the previously implanted and perforated temporary pacing wire. The latter was pulled during the LP implantation procedure, causing the pericardial effusion. These findings underscore the safety of the LP device implantation procedure itself.

Previous studies with smaller patient populations reported good performance of LP after TAVR, as well as other valve interventions.^17^, ^18^, ^19^, ^20^ Similarly, in our study, average pacing threshold, ventricular sensing, and impedance were within the target range at implantation and last follow-up, again without a difference between the TAVR and the non-TAVR groups, confirming that the LP in the TAVR group provided good-quality and stable performance.

### Important aspects to consider, and potential advantages of leadless pacing post-TAVR

Careful patient selection is crucial to achieving a high level of efficacy and safety of the procedure, as well as satisfying device performance overtime. The most recent pacing guidelines from the European Society of Cardiology (ESC) suggest that LP implantation should be considered when the risk of device pocket infection is particularly high (IIa, B) and that it may be considered as a general alternative considering life expectancy (IIb, C).^21^ Indeed, patients undergoing TAVR are often elderly and frail, which would merit the potential benefits of leadless pacing. Eliminating the need of a device pocket and transvenous leads, the major complication rate 2 years after implantation is significantly lower following LP implantation, compared to the rate with a historical transvenous pacemaker cohort.^22^ Most likely, this difference also will translate into a lower incidence of long-term complications, including device infections beyond 2 years.^23^^,^^24^ LP implantation after TAVR also has been demonstrated to permit a shorter hospital stay, which may reduce the risk of nosocomial infections and delirium in elderly and frail patients.^25^

Taking into account the rapid progress in both these arenas—pacing options as well as major advancements in TAVR procedures, an important point to consider is that indications for LP should evolve in tandem with the changing approach to TAVR. With TAVR being performed increasingly in a younger and healthier patient population, the appropriateness of an LP must be carefully evaluated, as traditional pacing options may remain favourable in these cases, with the option of upfront conduction system pacing instead of RV pacing. The relative ease of achieving left bundle branch pacing underscores the potential benefits of preserving the native conduction system, which may lead to improved cardiac synchrony and clinical outcomes. An important point to mention is that the longevity of the LP is shorter than that of a conventional transvenous system, and use of an LP most likely would require consecutive devices to be implanted in the right ventricle, as extraction is usually not performed at the time of implantation. On the other hand, successful LP extractions have been reported.

Furthermore, additional clinical baseline characteristics that have been associated with an increased risk of pacemaker-induced cardiomyopathy include a wider intrinsic QRS complex on electrocardiogram prior to device implantation and an anticipated > 40% RV pacing burden. Even in patients with normal LVEF, chronic RV pacing can lead to heart failure with impaired ejection fraction. An important point to stress is that, so far, LP implantation only allows RV pacing, limiting the use of an LP in patients with heart failure and reduced ejection fraction if a high pacing burden can be anticipated. A transvenous pacemaker offers the possibility of a later upgrade to a biventricular pacemaker or primary conduction system pacing to tackle this problem, both of which are not yet possible with an LP.^26^

In addition, in patients with a narrow QRS immediately post-TAVR, the risk of complete AV block is low.^27^ On the other hand, conduction disorders, such as left bundle branch block, are not uncommon, and in a number of cases, permanent pacing is chosen as a treatment strategy.^28^

Nonetheless, left ventricular outflow tract calcifications are associated with transient and nonreversible AV block post-TAVR, and their presence predicts a higher risk for need of permanent pacing.^29^ Also, patients requiring permanent pacing post-TAVR are known to have a higher long-term risk of all-cause mortality.^30^ As technology advances, additional patient monitoring before and after TAVR can be favoured to identify those with a potential increased risk for high-degree AV block, to address the question of what is the optimal treatment strategy.^31^^,^^32^

In summary, decisions for or against LP in patients after TAVR should incorporate consideration of patient-specific factors (ie, age, weight, chronic kidney disease, heart disease, LVEF, immunodeficiency, previous infections, vascular obstructions, sports activity, etc.), and careful shared decision-making in conjunction with the patient. Indeed, our cohort of post-TAVR patients was deemed to be frail elderly patients with multiple comorbidities in need of single-chamber pacing. Thus, our study suggests that LP implantation offers excellent acute and midterm results in this elderly and frail population who have an indication for VVI (V - pacing in the ventricle; V - sensing in the ventricle; I - inhibit) pacing.

On the other hand, introduction of the MICRA AV system with AV synchrony offers more physiological VDD (V - pacing in the ventricle; D sensing in the atrium and ventricle; D - inhibit and/or trigger) pacing and extends indications for an LP in patients with sinus rhythm, and the anticipated next AVEIR DR dual-chamber pacing system (Abbott, USA) will allow leadless DDD (D - pacing in the atrium and ventricle; D - sensing in the atrium and ventricle; D - inhibit and/or trigger) pacing.

In conclusion, if leadless pacing is preferred, it offers an efficient and safe pacing alternative after TAVR, according to the present data in this registry.

### Study limitations

The small number of patients, the retrospective design, referral bias, and selection bias are the main limitations of this study. For a coherent statistical analysis of significance, larger multicentre studies should be consulted. The operators were highly experienced, and were from 2 high-volume centres in Switzerland; hence, they may not be representative of other healthcare settings.
